# Unravelling the Complex Antimicrobial Interactions of Essential Oils — The Case of *Thymus vulgaris* (Thyme)

**DOI:** 10.3390/molecules19032896

**Published:** 2014-03-06

**Authors:** Aijaz Ahmad, Sandy van Vuuren, Alvaro Viljoen

**Affiliations:** 1 Department of Pharmaceutical Sciences, Tshwane University of Technology, Private Bag X680, Pretoria 0001, South Africa; E-Mail: ahmadA@tut.ac.za; 2 Department of Pharmacy and Pharmacology, University of Witwatersrand, 7 York Road, Parktown 2193, South Africa; E-Mail: Sandy.Vanvuuren@wits.ac.za

**Keywords:** antimicrobial activity, additivity, synergism, essential oil, *Thymus vulgaris*

## Abstract

*Thymus vulgaris* has gained tremendous popularity as an ornamental, culinary herb and its use in phytotherapy is well established and supported in the literature. The objective of this study was to explore possible interactions between selected molecules within *Thymus vulgaris* essential oil (TvEO) to gain a better understanding of how this complex essential oil exerts its antimicrobial activity. Evaluation of the antimicrobial efficacy and interactions were assessed on the essential oil and volatile constituents against various pathogens. Interactions between molecules at various ratios were graphically observed through the construction of isobolograms. Gas chromatography-mass spectrometry (GC-MS) analysis revealed 22 compounds which collectively represent >95% of the oil composition. Based on their minimum inhibitory concentration (MIC) values, they were categorised into weak (≥4 mg mL^−1^), moderate (2–4 mg mL^−1^) and noteworthy active (≤2 mg mL^−1^) compounds. For the combination study, 21% synergistic, 42% additive, 36% indifferent and 1% antagonistic interactions were observed. Most of the interactions were observed between the weak and highly active molecules, and interestingly, no synergistic interaction was observed between the highly active compounds. Synergistic and additive interactions between the strong and weaker antimicrobial constituents present in TvEO enhance the antimicrobial efficacy of this commercially important essential oil.

## 1. Introduction

The onset of drug resistance in the 21st century has reached a critical stage. In an attempt to combat several resistant strains, multi-drug target therapy has gained popularity [1]. Essential oils and their constituents are known to exhibit antimicrobial activity [2]. Research progress in complementary therapies has provided new insights into the use of essential oil constituents in combinations to treat and prevent infectious diseases [3]. The oil from *Thymus vulgaris*, a common culinary aromatic herb, ranks as one of the most intensively studied essential oils for its antimicrobial properties. The use of thyme dates back to the Roman era and it is reported that when the Black Death plague struck in the 1340s when many people used thyme to protect them from this devastating pandemic. Today, thyme has become a household name, as it is one of the most common herbs used to impart a specific flavour and fragrance in cooking. In addition, thyme oil is widely used in phytotherapy, most notably to treat and offer protection from acne, hypertension, infections and cancers [4]. The oil contains bioactive monoterpenes such as thymol, carvacrol and linalool. There are numerous studies dedicated to the antimicrobial activity of *Thymus vulgaris* essential oil (TvEO) as well as to its individual volatile constituents [5,6,7]. Most of these studies acutely focus on the antimicrobial activity of the crude essential oil and its major volatiles or possible synergistic interactions with conventional drugs against various pathogens. However, to the best of our knowledge, there is no systemic study on the possible interactions between individual molecules of this commercially important spice and household remedy. Our previous studies have shown significant synergistic antimicrobial interactions between volatile molecules [8,9]. Thus the objective of this study was to examine antimicrobial interactions between several constituents of TvEO and to specifically determine the contribution of the less active antimicrobial molecules of TvEO to enhance the antimicrobial activity.

## 2. Results and Discussion

### 2.1. Essential Oil Composition

**Figure 1:**
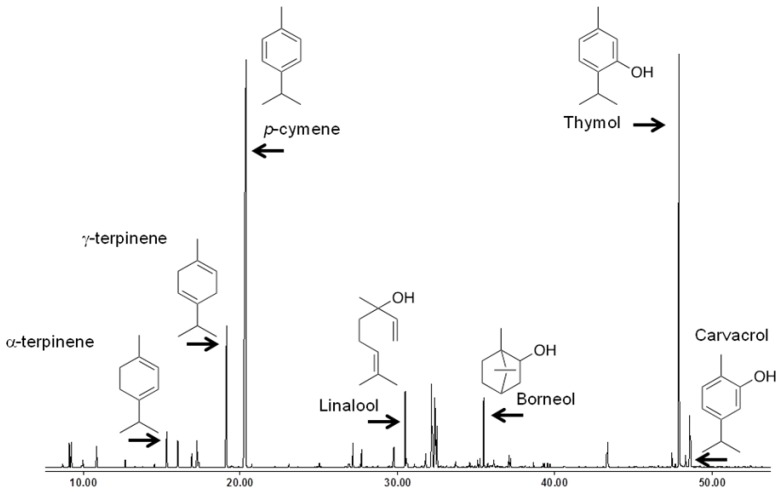
GC-MS analysis of *Thymus vulgaris* essential oil.

The GC-MS analysis of TvEO was undertaken to confirm the specific chemotype as *Thymus vulgaris* is considered as one of the best examples of chemotypic variation with seven different distinguishable monoterpenes occurring in various ratios [10,11]. Twenty two compounds representing <95% of the oil composition were identified (Figure 1). The TvEO used in the present study represents the thymol chemotype where the two major components comprise of thymol (60.18%) and *p*-cymene (15.44%) (Table 1), which is congruent with previously published profiles [5,12].

### 2.2. Minimum Inhibitory Concentrations (MIC)

Evaluation of the MIC values by broth microdilution assay showed that TvEO and selected molecules were active *in vitro* against Gram-positive, Gram-negative and yeast pathogens (Table 2). TvEO possessed high antimicrobial efficacy against all the tested pathogens with MIC values ranging from 0.062 to 0.500 mg mL^−1^. Based on commercial availability and structural chemical diversity selected molecules (thymol, *p*-cymene, γ-terpinene, linalool, carvacrol, borneol and α-terpinene) were tested for their specific contribution to the antimicrobial activity of the oil against the panel of pathogens (Table 2). 

**Table 1:** Chemical composition of *Thymus vulgaris* essential oil.

Compounds	RRI	% of oil
α-Pinene	1016	0.63
α-Thujene	1019	0.50
Camphene	1057	0.61
β-Pinene	1104	0.17
Myrcene	1159	1.17
α-Terpinene	1174	1.01
Limonene	1194	0.35
1,8 Cineole	1202	0.24
β-Phellandrene	1203	0.13
γ-Terpinene	1242	6.39
p-Cymene	1270	15.44
Camphor	1521	0.39
Linalool	1541	4.22
β-Caryophyllene	1596	1.31
Terpinen-4-ol	1602	0.93
Thymol methyl ether	1607	0.51
Boroneol	1702	1.76
α-Terpineol	1707	0.32
δ-Cadinene	1763	0.09
Isothymol	2171	0.12
Thymol	2225	60.18
Carvacrol	2228	2.88
Total:		99.35

**Table 2:** Minimum inhibitory concentrations of *Thymus vulgaris* essential oil and its major constituents when tested individually against a panel of pathogens.

Pathogen	Minimum Inhibitory Concentrations (mg mL^−1^)								
	TvEO	Thymol	Carvacrol	Linalool	*p*-Cymene	Borneol	α-Terpinene	γ-Terpinene	Controls *
*Escherichia coli* ATCC8739	0.500	1	1	4	>8	>8	8	>8	0.0005
*Morexella cattarhalis* ATCC23246	0.500	1	1	2	>8	8	8	>8	0.001
*Staphylococcus aureus* ATCC126000	0.500	1	0.500	2	>8	8	4	8	0.0005
*Enterococcus faecalis* ATCC29212	0.125	0.500	0.500	1	>8	4	8	>8	0.001
*Bacillus cereus*ATCC11778	0.125	0.500	0.250	2	>8	8	4	8	0.0002
*Candida albicans* ATCC10231	0.062	0.125	0.125	2	>8	1	1	8	0.001
*Candida tropicalis*ATCC201380	0.062	0.125	0.125	0.250	>8	1	0.250	4	0.001

Antimicrobial activity = Noteworthy activity (MIC < 2 mg mL^−1^); moderately active (MIC 2–4 mg mL^−1^); weakly active (MIC > 4 mg mL^−1^); * Controls are ciprofloxacin for bacteria and amphotericin B for the yeasts.

**Table 3:** The fractional inhibitory concentration index of selected compounds of *Thymus vulgaris* essential oil tested in 1:1 combinations.

EO compoundsmg mL^−1^				*E. coli*			INT			*M. cattarhalis*			INT			*S. aureus*			INT			*E. faecalis*				INT		*B. cereus*				INT		*C. albicans*				INT		*C. tropicals*			INT
**THY + CARV**	FIC_A_			0.500			ADD			0.500			ADD			1.000			IND			1.000				IND		1.000				IND		0.500				ADD		0.500			ADD
	FIC_B_			0.500						0.500						2.000						1.000						2.000						0.500						0.500			
	∑FIC			1.000						1.000						3.000						2.000						3.000						1.000						1.000			
**THY + LIN**	FIC_A_			0.500			ADD			1.000			IND			0.500			ADD			0.500				ADD		2.000				IND		0.500				ADD		0.500			ADD
	FIC_B_			0.250						0.500						0.250						0.250						0.500						0.031						0.250			
	∑FIC			0.750						1.500						0.750						0.750						2.500						0.531						0.750			
**THY + CYM**	FIC_A_			1.000			IND			0.250			**SYN**			0.250			**SYN**			1.000				IND		1.000				IND		0.500				**SYN**		0.500			**SYN**
	FIC_B_			0.063						0.031						0.031						0.031						0.031						0.004						0.004			
	∑FIC			1.063						**0.281**						**0.281**						1.031						1.031						**0.504**						**0.504**			
**THY + BOR**	FIC_A_			1.000			IND			4.000			ANT			0.500			ADD			2.000				IND		0.250				**SYN**		0.500				ADD		0.500			ADD
	FIC_B_			0.063						1.000						0.062						0.250						0.016						0.063						0.250			
	ƩFIC			1.063						5.000						0.562						2.250						**0.261**						0.563						0.750			
**THY + α-TER**	FIC_A_			1.000			IND			0.500			ADD			1.000			IND			1.000				IND		0.500				ADD		2.000				IND		0.500			ADD
	FIC_B_			0.120						0.063						0.250						0.063						0.063						0.250						0.250			
	ƩFIC			1.125						0.563						1.250						1.063						0.563						2.250						0.750			
**THY + γ -TER**	FIC_A_			1.000			IND			1.000			IND			1.000			IND			2.000				IND		2.000				IND		1.000				IND		1.000			IND
	FIC_B_			0.063						0.063						0.125						0.063						0.125						0.015						0.031			
	∑FIC			1.063						1.063						1.125						2.063						2.125						1.015						1.031			
**CARV + LIN**	FIC_A_			0.500			ADD			0.500			ADD			0.500			ADD			1.000				IND		0.250				**SYN**		0.500				ADD		0.500			ADD
	FIC_B_			0.250						0.250						0.125						0.500						0.030						0.031						0.250			
	ƩFIC			0.750						0.750						0.625						1.500						0.281						0.531						0.750			
**CARV + CYM**	FIC_A_			0.125			**SYN**			0.125			**SYN**			0.500			**SYN**			0.500				**SYN**		0.250				**SYN**		1.000				IND		1.000			IND
	FIC_B_			0.032						0.008						0.008						0.008						0.008						0.008						0.008			
	∑FIC			**0.157**						**0.133**						**0.508**						**0.508**						**0.258**						1.008						1.008			
**CARV + BOR**	FIC_A_			2.000			IND			0.500			ADD			0.250			**SYN**			0.500				ADD		0.500				ADD		0.500				ADD		0.500			ADD
	FIC_B_			0.125						0.125						0.015						0.125						0.016						0.063						0.250			
	ƩFIC			2.125						0.625						**0.265**						0.625						0.516						0.563						0.750			
**α-TER + CARV**	FIC_A_			0.500			ADD			0.500			ADD			0.250			**SYN**			1.000				IND		0.250				**SYN**		0.250				**SYN**		0.500			ADD
	FIC_B_			0.625						0.063						0.062						0.063						0.250						0.063						0.250			
	FIC			0.562						0.563						**0.312**						1.063						**0.500**						**0.313**						0.750			
**CARV + ** **γ -TER**	FIC_A_			0.500			ADD			0.500			ADD			0.250			ANT			1.000				IND		0.500				ADD		0.250				**SYN**		0.500			ADD
	FIC_B_			0.063						0.031						4.000						0.031						0.030						0.015						0.015			
	ƩFIC			0.563						0.531						4.250						1.031						0.530						**0.265**						0.515			
**LIN + CYM**	FIC_A_			2.000			IND			0.250			**SYN**			1.000			IND			0.500				ADD		1.000				IND		1.000				IND		0.125			**SYN**
	FIC_B_			0.250						0.062						0.125						0.063						0.125						0.125						0.016			
	FIC			2.250						**0.312**						1.125						0.563						1.125						1.125						**0.141**			
**LIN + BOR**	FIC_A_			0.500			ADD			O.500			ADD			0.250			**SYN**			0.500				ADD		0.250				**SYN**		0.250				ADD		1.000			IND	
	FIC_B_			0.125						0.250						0.062						0.125						0.063						0.500						1.000				
	ƩFIC			0.625						0.750						**0.312**						0.625						**0.312**						0.750						2.000				
**LIN +α-TER**	FIC_A_			0.250			**SYN**			0.500			ADD			1.000			IND			0.250				**SYN**		0.500				ADD		0.500				IND		0.250			**SYN**	
	FIC_B_			0.063						0.125						0.500						0.031						0.250						1.000						0.250				
	ƩFIC			**0.312**						0.625						1.500						**0.281**						0.750						1.500						**0.500**				
**LIN + γ -TER**	FIC_A_			1.000			IND			0.500			ADD			1.000			IND			2.000				IND		0.500				ADD		0.500				ADD		1.000			IND	
	FIC_B_			0.120						0.063						0.250						0.125						0.125						0.125						0.063				
	ƩFIC			1.120						0.563						1.250						2.125						0.625						0.625						1.063				
**CYM + BOR**	FIC_A_			0.500			ADD			0.063			**SYN**			0.250			**SYN**			0.250				IND		0.250				ADD		0.125				IND		1.000			IND	
	FIC_B_			0.500						0.125						0.125						1.000						0.500						2.000						0.015				
	ƩFIC			1.00						**0.188**						**0.375**						1.250						0.750						2.125						1.015				
**CYM + α-TER**	FIC_A_			0.500			IND			0.250			ADD			0.500			IND			0.250				ADD		0.250				IND		0.125				IND		1.000			IND	
	FIC_B_			1.000						0.500						2.000						0.500						1.000						2.000						0.015				
	ƩFIC			1.500						0.750						2.500						0.750						1.250						2.125						1.015				
**CYM + ** **γ -TER**	FIC_A_			0.500			ADD			0.063			**SYN**			0.500			IND			0.500				ADD		0.250				ADD		0.500				IND		0.500			IND	
	FIC_B_			0.500						0.125						1.000						0.500						0.500						1.000						2.000				
	ƩFIC			1.000						**0.188**						1.500						1.000						0.750						1.500						2.500				
**BOR + ** **α-TER**	FIC_A_			0.250			**SYN**			0.500			ADD			0.250			ADD			0.500				ADD		0.500				IND		0.500				ADD		0.124			**SYN**	
	FIC_B_			0.250						0.250						0.500						0.250						1.000						0.500						0.124				
	ƩFIC			**0.500**						0.750						0.750						0.750						1.500						1.000						**0.248**				
**BOR + γ -TER**	FIC_A_			0.250			**SYN**			0.500			ADD			0.500			ADD			0.500				ADD		0.500				ADD		0.250				**SYN**		2.000			IND	
	FIC_B_			0.250						0.250						0.500						0.125						0.500						0.031						0.125				
	ƩFIC			**0.500**						0.750						1.000						0.625						1.000						**0.281**						2.125				
**α-TER + γ -TER**	FIC_A_			0.500			ADD			0.500			ADD			0.500			ADD			0.125				**SYN**		0.500				ADD		1.000				IND		1.000			IND	
	FIC_B_			0.250						0.250						0.250						0.125						0.250						0.125						0.016				
	ƩFIC			0.750						0.750						0.750						**0.250**						0.750						1.125						1.016				

INT: Interpretation, ADD: additive, SYN: synergy, IND: indifferent, ANT: antagonism, THY: thymol, CARV: carvacrol, LIN: linalool, CYM: p-cymene, BOR: boroneol, γ –TER: γ-terepinen, α-TER: α-terepinen, FIC_A_: MIC of compound A when combined with compound B, FIC_B_: MIC of compound B when combined with compound A.

Based on these MIC values, the test compounds were categorised into three groups: noteworthy (MIC ≤ 2 mg mL^−1^), moderatly active (MIC 2–4 mg mL^−1^) and weakly active compounds (MIC ≥ 4 mg mL^−1^). As expected, the monoterpene phenols, thymol and carvacrol, were found to be the most active constituents, while linalool were generally found to have moderate activity and *p*-cymene, borneol, α-terpinene and γ-terpinene exhibited weak antimicrobial activity (Table 2). The chromatographic profiling of TvEO confirmed the presence of two important groups, *i.e.*, monoterpene hydrocarbons and oxygenated monoterpenes as lead molecules. The former group is not an efficient antimicrobial class; conversely the later is known to possess high antimicrobial activities [13]. Therefore, terpenoids like thymol and carvacrol are the major contributors to the antimicrobial activity of TvEO. However, it is evident that the crude oil exhibits higher antimicrobial activity compared to individual (major) constituents suggesting the involvement of other components in the antimicrobial activity of this essential oil. This aspect was further explored and the antimicrobial activity of all major compounds alone and in combinations was determined to investigate potential synergistic interactions between the various constituents contributing to the overall efficacy of the TvEO.

**Figure 2:**
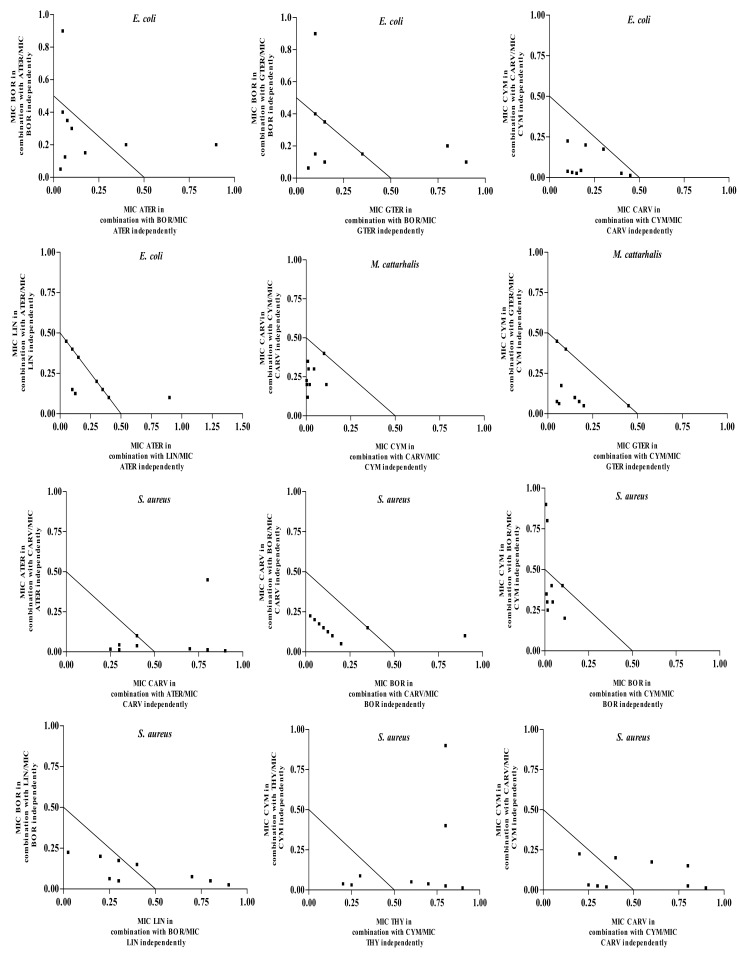

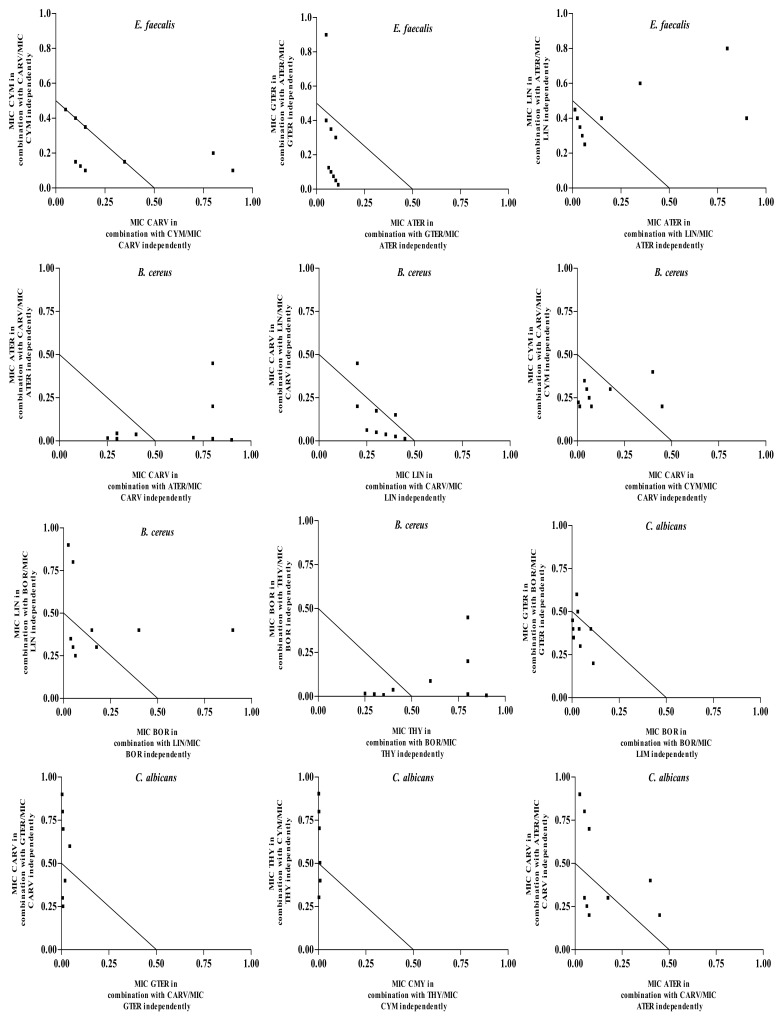

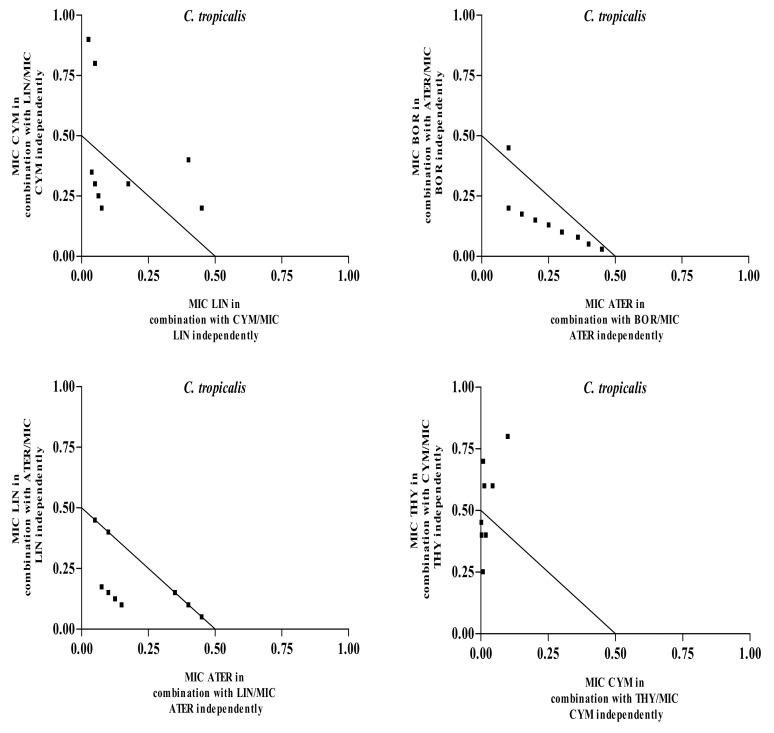
Isobolograms of active molecules in nine different ratios against selected pathogens.

To determine the possible combinational interactions of the selected molecules, fractional inhibitory concentration indices (FICI) were calculated using the microbroth dilution method as described previously [3,14]. The FICI values for seven major constituents in 1:1 ratios showed either synergistic, additive or indifferent interactions. Of the 147 combinations studied (Table 3), only two antagonistic interactions were observed, which was for the combination of thymol and borneol against *M. cattarhalis*, and the combination of γ-terpinene and carvacrol against *S. aureus*. The FICI of the assayed molecules against all the tested pathogens ranged from 0.133 to 5.000. Synergistic interactions were mostly observed with the Gram-positive micro-organisms (28%), particularly *S. aureus* and *B. cereus*, demonstrating eight synergistic interactions each. The yeasts and Gram-negative strains followed, showing 18% and 14% synergistic interactions respectively. For all the combinations tested (*n* = 147), 21% were found to be synergistic, 42% additive, 36% indifferent and 1% antagonistic (Table 3). The most pronounced synergistic interaction was observed between the weakly active *p*-cymene (an alkyl benzene monoterpene) and the strongly active carvacrol (a cyclic monoterpene phenol) against *M. cattarhalis* (FICI = 0.133). Also a pronounced synergistic interaction was also observed between *p*-cymene and linalool (FICI = 0.141) and the combination *p*-cymene and carvacrol (FICI = 0.157) against *C. tropicalis* and *E. coli*, respectively. It is interesting to observe that most of these prominent synergistic interactions are between compounds, which showed strong and weak antimicrobial activity when tested singularly. From the results it is clearly evident that out of all the synergistic interactions, 48% is between the compounds with weak and strong antimicrobial activities when tested alone. When compounds exhibiting weak MICs were combined in a 1:1 ratio, 26% of tested combinations showed synergy. A good example is the combination of thymol (stronger antimicrobial) with the weaker *p*-cymene where synergistic interactions were noted against four pathogens. Similarly, the carvacrol and *p*-cymene combination showed synergy against five of the seven pathogens studied. It is often assumed that only strongly active compounds contribute towards the overall activity of the whole oil. These results demonstrate that highly active compounds together with compounds with poorer activity have an additive and even synergistic effect. For twenty eight of the synergistic combinations observed in the 1:1 combination, further in-depth studies were carried out through the construction of isobolograms. Molecules rarely occur in a one-to-one ratio in a crude oil or herbal extract. Isobolograms were thus plotted to demonstrate the antimicrobial effects of a range of differently combined ratios of the compounds that demonstrated synergy in the 1:1 combinations. Nine different ratios for each synergistic combination were mixed and antimicrobial efficacies were determined (Figure 2).

From the isobolograms it is evident that most of the combinations, regardless of the ratio, display synergistic interactions. When synergy wasn’t observed, additivity was apparent and interestingly no antagonism, nor non-activity was observed for any of the ratios. This clearly indicates how all these compounds interact, which could explain efficacies in other oils having similar molecules. The combinations of *p*-cymene and carvacrol against *E. coli* and *M. cattarhalis* showed synergistic interactions for all nine ratios studied. When the same combination was assayed against *B. cereus* and *E. faecalis* seven out of nine ratios showed synergy, while two of the ratios exhibited additive effects. Terpinenes such as *p*-cymene, α-terpinene and γ-terpinene when tested alone were found to be weakly active against both bacterial and fungal pathogens [15,16,17]. Our results are in agreement with these findings. However, when *p*-cymene is paired with carvacrol or thymol, 64% of the combinations were synergistic. *p*-Cymene, a precursor molecule of carvacrol and thymol, is hypothesized as a substitutional impurity in the membrane causing membrane expansions by decreasing the enthalpy and melting temperatures of the membranes without disturbing the permeability [18]. This expansion of membranes by *p*-cymene possibly potentiates the activity of other molecules to disrupt the membranes and penetrate the cell to interact with the intracellular drug targets leading to cell death. *p*-Cymene was also found to be a common denominator in synergistic interactions when combined with linalool, γ-terpinene and borneol. 

The combination of γ-terpinene and α-terpinene with borneol against *C. albicans* and *C. tropicalis* showed synergistic interactions for seven and eight ratios, respectively, while at the other ratios an additive response was observed. With these combinations, it was also noted that γ-terpinene or α-terpinene is present in the higher ratio synergistic interactions. Both γ-terpinene and α-terpinene are cyclic monoterpenes known to disrupt the membrane lipid bilayers resulting in lipid leakages [19], which might promote the passage of other molecules (e.g., bicyclic borneol) into the cell resulting in the enhancement of their antimicrobial efficacy. 

A solid body of evidence exists illustrating the specific mode of action of natural products. Thyme oil contains two potent monoterpene phenols, thymol and carvacrol, which exert their antimicrobial action by disruption and depolarisation of the cytoplasmic membranes and by targeting membrane proteins and intracellular drug targets [13,20]. It has also been observed that these terpenoids have delocalised electrons which facilitates the dissociation of H^+^ from the -OH group leading to the dissipation of pH and ion gradients across the membrane [21]. It has been confirmed that terpinenes (e.g., γ-terpinene and α-terpinene) act as vehicles which enhance the influx of other compounds to reach their intracellular drug targets. Both terpinenes and terpenoids are known to cause membrane instability; however, it has been shown that terpinenes when combined with terpenoids increase the number and size of the pores leading to synergistic interactions between the two classes of molecules [13,18].

## 3. Experimental

### 3.1. Strains, Media and Chemicals

All strains (Table 2) were initially grown in Tryptone Soya broth (TSB). Pure cultures were thereafter maintained on Tryptone Soya agar (TSA) plates and were sub-cultured and incubated for their respective incubation periods at 37 °C prior to assays. The essential oil of *Thymus vulgaris* was kindly supplied by Robertet Ltd (Paris, France, EO853). All the essential oil standards were purchased from Sigma Aldrich (St. Louis, MO, USA); ciprofloxacin (CFL) and amphotericin B (AmB) were obtained from Sigma Fluka (St. Louis, MO, USA). All other chemicals and media were of analytical grade and were procured from Oxide Ltd. (Basingstoke, Hampshire, England).

### 3.2. Gas Chromatography-Mass Spectrometry Analysis

*Thymus vulgaris* essential oil (TvEO) was subjected to GC-MS analysis using a gas chromatograph coupled to a mass spectrometer and flame ionization detector (GCMS-FID) as described previously [3]. The Agilent (6890N) GC system (Agilent Technologies, Inc, Santa Clara, CA, USA) was equipped with a HP-Innowax polyethylene glycol column (60 m × 250 μmi.d × 0.25 μm film thickness). The chemical components were identified by comparing mass spectra from the total ion chromatogram, and retention indices using NIST and Mass Finder GC-MS libraries.

### 3.3. Antimicrobial Susceptibility Tests

#### 3.3.1. Determination of Minimal Inhibitory Concentration (MIC)

The MICs of TvEO and selected molecules were determined by the broth microdilution method as approved by the guidelines of Clinical and Laboratory Standards Institute [22]. Both essential oil and essential oil standards were diluted to 32 mg mL^−1^ using acetone as a diluent. The microtitre plates were prepared by adding 0.1 mL of TSB into each of the wells followed by an addition of the test substance at a volume of 0.1 mL (when tested individually) and 0.05:0.05 mL (when tested in combination). The test compounds were serially diluted to yield concentrations of 8, 4, 2, 1, 0.5, 0.25, 0.125, and 0.0625 mg mL^−1^. The positive control ciprofloxacin (0.01 mg mL^−1^) for bacteria and amphotericin B (0.1 mg mL^−1^) for yeasts and the negative vehicle control (acetone/water solution 32 mg mL^−1^) were also included in every set of experiments. Media and culture controls were included to confirm the sterility and viability, respectively. The reference strain test organisms (Table 2), with an approximate final inoculum size of 1 × 10^6^ colony forming units (CFU) ml^−1^, were then added to each well, at a volume of 0.1 mL. The microtitre plates were sealed with a sterile adhesive film, to prevent any essential oil loss due to their inherent volatility. The microtitre plates were incubated under optimal conditions (37 °C for 24 h for bacteria and 37 °C for 48 h for yeasts). After incubation, 0.4 mg mL^−1^ of *p-*iodonitrotetrazolium violet solution (INT) was added to each well (0.04 mL). Viable micro-organisms interact with INT to create a colour change from clear to a red-purple colour. Thus the lowest dilution with no colour change was considered as the MIC for that test compound or TvEO [3]. All the results were calculated as a mean of the experiments done in duplicate.

#### 3.3.2. Assessment of the FIC Index

To determine the interactions of the selected individual molecules of the TvEO, microdilution assays were performed in 96-well microtitre plates as described previously [3]. Briefly, 1:1 volumes (50 µL; 50 µL) of the standard molecules were added to the microtitre plates with 0.1 mL media and serially diluted as described in the MIC methodology. All the controls as described for the MIC determination were also included in this study. To assess the interactions, the data obtained were further analysed using the fractional inhibitory concentration index (FICI), which is based on the zero-interaction theory of Loewe additivity [23]. FICI was defined as:

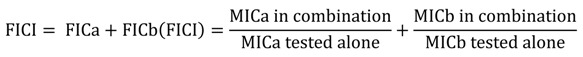
(1)
The MIC_a_ and MIC_b_ are the MICs of the compounds (thymol, carvacrol, linalool, *p*-cymene, borneol, α-terpninene and γ-terpinene). An FICI value was interpreted as synergy where the FICI is ≤0.5 and antagonism where the FICI is >4. An FICI result between 0.5 and 1.0 was considered additive and a value between 1.0 and 4.0 was considered as indifferent [24,25].

#### 3.3.3. Varied Ratio Combinations and Isobolograms

On the basis of the promising synergistic interactions observed in the microdilution assay, isobolograms were constructed. Nine ratios (9:1; 8:2; 7:3; 6:4; 5:5; 4:6; 3:7; 2:8; and 1:9) of the essential oil compounds were mixed and thereafter the MIC values were determined for these combinations, as well as for the test compounds independently. Isobolograms were plotted using GraphPad Prism, version 5 software (GraphPad Software, Inc. California, CA, USA), to present the mean MIC values of the combinations as ratios [26]. The isobolograms were interpreted by examining the data points for each ratio in relation to the MIC values for the oils independently. All points between the 1.0:1.0 line and 4.0:4.0 line were classified as non-interactive. Points between the 0.5:0.5 and 1.0:1.0 line were interpreted as additive and points below or on the 0.5:0.5 line on the isobologram were interpreted as synergistic. Antagonism was identified as data points above the 4.0:4.0 line [25].

## 4. Conclusions

The literature is flooded with papers reporting the antimicrobial activity of botanical extracts and isolated natural products. Fewer papers, however, attempt to unravel the potential interaction between molecules, which will inevitably lead to a better understanding of the observed antimicrobial properties. In this study we have used a well-known plant to illustrate some of the fascinating, yet complex interactions which collectively contribute to the activity of a crude essential oil. Many of the essential oil compounds have different modes of actions and therefore, when used in combination, they reduce the concentration needed to achieve an antimicrobial effect. By our own admission, this study simply presents a snapshot of the intricate interactions in Thyme oil, as it would be an impossible task to explore all the possibilities. Further in-depth studies of the modes of action for synergistic combinations are encouraged. We have reported 22 compounds in thyme oil (ignoring the presence of very minor compounds and enantiomers). Theoretically this implies that 4,194,303 combinations exist which could be tested, clearly a Herculean task. This study, however, has shown the complex interactions between several essential oil constituents with many of these combinations being synergistic confirming that synergy is the very premise on which the concept of phytotherapy is based.
